# The Neural Correlates of Probabilistic Classification Learning in Obsessive-Compulsive Disorder: A Pilot Study

**DOI:** 10.3389/fpsyt.2018.00058

**Published:** 2018-02-28

**Authors:** Jana Hansmeier, Cornelia Exner, Ulrike Zetsche, Andreas Jansen

**Affiliations:** ^1^Division of Clinical Psychology and Psychotherapy, Philipps University of Marburg, Marburg, Germany; ^2^Department of Clinical Psychology, University of Leipzig, Leipzig, Germany; ^3^Department of Clinical Psychology, Freie Universität Berlin, Berlin, Germany; ^4^Department of Psychiatry and Psychotherapy, Philipps University of Marburg, Marburg, Germany; ^5^Core-Unit Brainimaging, Faculty of Medicine, Philipps University of Marburg, Marburg, Germany; ^6^Marburg Center for Mind, Brain and Behavior (MCMBB), Marburg, Germany

**Keywords:** obsessive-compulsive disorder, probabilistic classification learning, implicit learning, hippocampus, striatum

## Abstract

Individuals suffering from obsessive-compulsive disorder (OCD) have been found to show deficits in implicitly learning probabilistic associations between events. Neuroimaging studies have associated these implicit learning deficits in OCD individuals with aberrant activation of the striatal system. Recent behavioral studies have highlighted that probabilistic classification learning (PCL) deficits in OCD individuals only occur in a disorder-specific context, while PCL remains intact in a neutral context. The neural correlates of implicit learning in an OCD-specific context, however, have not yet been investigated. Using functional magnetic resonance imaging during a neutral (prediction of weather) and an OCD-specific variant (prediction of a virus epidemic) of a PCL paradigm, we assessed brain activity associated with implicit learning processes in 10 participants with OCD and 10 matched healthy controls. Regions of interest (ROIs) were the striatum and the medial temporal lobe. ROI analyses revealed a significantly higher activity in the bilateral putamen and the left hippocampus of OCD participants as compared to healthy controls during both PCL tasks. The group differences could partly be subsumed under a group × task interaction effect with OCD participants showing a significantly higher activity than healthy controls in the left putamen and the left hippocampus in the OCD-specific task variant only. These results suggest a compensation of aberrant striatal activity by an augmented engagement of the explicit memory system particularly in a disorder-relevant context in OCD participants.

## Introduction

The tendency to overestimate the probability and consequences of threatening events has been proposed to play a role in the etiology and maintenance of obsessive-compulsive disorder (OCD) (1). Indeed, studies have found an elevated tendency to overestimate threat not only in individuals with OCD (2) but also in their healthy relatives (compared to unrelated healthy controls) (3, 4). The tendency to overestimate threat in OCD individuals might be associated with difficulty in learning cue–outcome associations that are linked in a probabilistic manner (e.g., likelihood of being infected after using a public toilet). The cognitive ability to build these associations relies on implicit information processing. Several studies have suggested that participants with OCD show a general deficit in implicit learning of probabilistic associations between cues and outcomes (5–9).

Neurobiological models of OCD participants posit that the disorder is associated with dysfunctions in the orbitofrontal–striatal system [e.g., Ref. (10)]. In line with this proposition, structural and functional abnormalities are frequently reported in the orbitofrontal cortex and the caudate nucleus in OCD participants [e.g., Ref. (11–13)]. Striatal circuits, however, are also assumed to be the neural basis of implicit learning. For example, the caudate nucleus and the cortical areas projecting to striatal circuits are activated during implicit learning tasks (14–16). In addition, Rauch et al. (17) have shown that striatal and thalamic areas are activated during implicit learning.

To sum up, these findings suggest deficits in implicit learning in OCD participants which may be based on dysfunctions in the striatal circuits. However, behavioral studies of implicit learning in OCD participants have produced mixed results. Several studies found deficits in implicit learning in OCD participants (5–9), whereas other studies showed that individuals with OCD did not differ from healthy controls in their implicit learning performance (18), or even showed a better performance than healthy controls (19). To better understand these diverging results, it may be important to understand the neurobiological correlates of impaired and preserved implicit learning in OCD participants.

Recent neuroimaging studies of implicit learning in the serial reaction time task (SRT) in OCD have demonstrated that individuals with OCD showed unexpected activation of the medial temporal lobe including the hippocampus, whereas healthy controls only showed striatal activation (20, 21). The activation of the medial temporal lobe (22) might indicate that participants with OCD, similar to patients with Parkinson’s disease (23), compensate for possible striatal dysfunction by engaging the explicit learning system. This finding is in line with that obtained in a previous study (8) showing that implicit learning is disrupted in participants with OCD when a concurrent explicit task prevents use of compensatory processes.

An important question being unanswered so far is how the possible striatal deficits come into effect when implicit learning takes place in a disorder-specific context. Considering that individuals with OCD perceive themselves as more vulnerable to experience OCD-related events (24, 25) and show a fronto-striatal activation particularly in the context of symptom provocation (26–28), an investigation of implicit learning in a disorder-specific context seems to be especially relevant. In a previous study of our research group (18), we used two variants of a well-known probabilistic classification learning (PCL) (29): the original neutral variant and an adapted OCD-relevant version (prediction of the onset of an epidemic from virus infection). Results showed that participants with OCD performed as well as controls in the neutral task but scored significantly below healthy controls in the OCD-specific task. Using the same paradigm, two other studies found that the performance of OCD participants in the OCD-specific task was associated with biases in the prediction of checking-related events (30) and an inflexible strategy use (31). In healthy controls, arousal during encoding had a disruptive effect on implicit learning performance (32, 33) and was related to a suboptimal strategy use (34) in PCL. The neural correlate appearing to serve a critical function in the emotional modulation of memory retention is the amygdala [for a review, see Ref. (35)]. With regard to implicit learning, the presence of fear-relevant outcomes was related to reduced recruitment of the caudate nucleus and the amygdala in PCL (36).

Implicit learning in OCD participants has predominantly (and with regard to their neural correlates only) been investigated using the SRT paradigm so far. However, considering that the SRT involves high motor requirements and participants with OCD suffer more from uncontrollable, intrusive thoughts than from stereotypical behavioral routines, the performance in a non-motor implicit learning task like the PCL would be of particular interest. The present study therefore aimed to investigate the neural correlates of implicit learning in OCD participants using the PCL paradigm with a neutral and an OCD-specific task. Thus, we tested the following hypotheses:

Hypothesis 1: In the neutral PCL task, participants with OCD will show altered activation in the striatum and in the medial temporal lobe (particularly in the hippocampus) as compared to healthy controls, despite equivalent behavioral performance.
Hypothesis 2: In the OCD-specific PCL task, participants with OCD will show a lower performance as compared to healthy controls, which is related to altered activation of the striatum, the hippocampus and the amygdala.

## Materials and Methods

### Participants

The sample comprised 10 participants with OCD (6 female, 29.7 ± 7.6 years; see Table S1 in Supplementary Material). Participants were recruited for the study from outpatient clinics (*n* = 2), by advertisements in local newspapers and supermarkets (*n* = 6), and on an OCD web portal (*n* = 2). All participants met criteria for current OCD according to the Diagnostic and Statistical Manual of Mental Disorders [DSM-IV; see Ref. (37)]. The diagnosis was verified by SCID interview (see Clinical Assessment). Eight participants with OCD presented with one to two comorbid current mental disorders (major depressive disorder or dysthymia in four participants, anxiety disorders in five participants, and eating disorders in two participants). Subjects with a history of head injury, neurological diseases, psychoses, or substance dependence were excluded. Six participants with OCD were on psychotropic medication (selective serotonin reuptake inhibitors or other antidepressant agents, one with a mood stabilizer, one with an atypical neuroleptic). The dimension of OC symptoms with the highest mean score (as measured by the Padua Inventory—Washington State University Revision) in the OCD group was contamination obsessions and washing compulsions (M = 2.9, SD = 1.4), followed by checking compulsions (M = 2.5, SD = 1.1), dressing/grooming compulsions (M = 2.0, SD = 1.4), obsessional thoughts of harm to self/others (M = 1.9, SD = 1.0), and obsessional impulses to harm self/others (M = 1.3, SD = 0.44).

Participants with OCD were compared with 10 healthy controls (6 female, 29.3 ± 8.6 years) recruited for the study by advertisements in local newspapers and leaflets distributed in town. Healthy controls were not taking any psychoactive medication and were free of any psychiatric disorder (verified by SCID interview), neurological disorder, and significant medical illness.

Healthy controls matched participants with OCD in terms of age, sex, years of education, and intelligence (see Table S1 in Supplementary Material). The clinical and demographic characteristics of participants are summarized in Table S1 in Supplementary Material.

### Clinical Assessment

The Ethical Committee of the German Psychological Society (DGPs) approved the study. Participants received a complete oral and written description of the study and provided written informed consent. A German version (38) of the Structured Clinical Interview for DSM-IV [SCID; see Ref. (39)] was administered by a trained psychologist to assess current and lifetime psychiatric diagnoses in both OCD participants and healthy controls. The SCID has demonstrated good reliability for various disorders (40). The interviewer had extensive training in the reliable use of the SCID. OC symptoms were rated in participants with OCD using the German-authorized translation (41) of the Yale–Brown Obsessive Compulsive Scale [Y-BOCS; see Ref. (42)] and the Padua Inventory–Washington State University Revision [PI-WSUR; see Ref. (43)]. Participants with OCD and healthy controls also completed the 44-item German version (44) of the Obsessive Beliefs Questionnaire [OBQ; see Ref. (1)]. Previous factor analyses of the OBQ have yielded different factor solutions, resulting in three to six factors (44–48). We used the six rationally derived scales (44, 48) in order to investigate the specific relation of these hypothesized domains of obsessive-compulsive beliefs to brain activity during PCL. Self-reports of depressive symptoms were obtained from the German version (49) of the Beck Depression Inventory [BDI; see Ref. (50)]. General trait anxiety was assessed with the trait scale of the State Trait Anxiety Inventory [STAI; see Ref. (51)], German version (52).

### Neuropsychological Assessment

In order to control for the influence of overall cognitive and explicit memory performance, a number of control tests were administered. The German version (53) of the Wechsler Adult Intelligence Scale—3rd edition [WAIS-III; see Ref. (54)] was applied to estimate intellectual functioning (subtests information, similarities, picture completion, and block design). Explicit episodic verbal memory was assessed with the subtests Logical Memory I and II and Verbal Paired Associates I and II from the German version (55) of the Wechsler Memory Scale—Revised [WMS-R; see Ref. (56)]. The two subtests Visual Paired Associates I and II from the same battery were administered to assess visual episodic memory.

### PCL and Control Condition

Two variants of a PCL task, which had been used in a previous behavioral investigation (18), were administered on a computer screen. In both tasks, participants learned to predict which of two future events would occur on each trial after presentation of a particular combination of one, two, or three visual cues (out of four possible cues). Each cue was independently associated with each of the two outcomes with a fixed probability (75, 57, 43, and 25%, respectively) and the two outcomes occurred equally often across the task. The probability structure was modeled after the original task by Knowlton et al. (29) and was identical for both tasks. Two sets of visual cues, which resembled pseudo-Chinese characters, were used counterbalanced across tasks.

**Figure 1:**
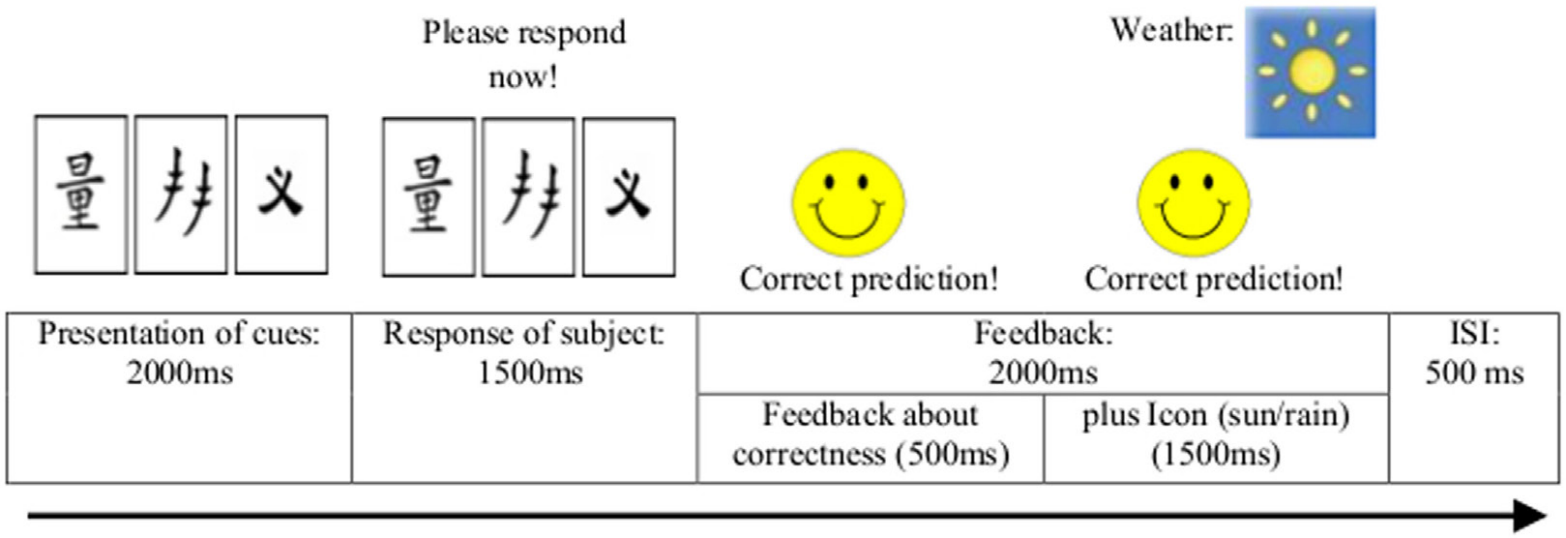
Sequence of events during each trial of the two probabilistic classification learning tasks.

The sequence of events across a trial is depicted in Figure 1. At the beginning of each trial, the visual cues appeared on the screen for 2 s. Participants indicated their choice by pressing one of two keys assigned to the two outcomes, for which they had 1.5 s time. Feedback was presented after the response and lasted 2 s. If the response was correct, a smiling face appeared at the left of the screen together with a verbal feedback line reading: “Correct prediction!”. If the response was incorrect, a frowning face appeared together with a verbal feedback line reading: “Incorrect prediction!”. In either case, the icon indicating the correct answer appeared on the right-hand side of the screen above the cues for 1.5 s. After an interstimulus interval of 0.5 s, the next cue pattern was presented. Thus, in either case, one trial lasted up to 6 s in total.

A cover story was given for each of the two tasks. In the neutral task variant, the cover story implied that the subject was situated in the local meteorological station and was receiving data on the weather condition from a new instrument, whose output had yet to be deciphered. Participants decided on each trial whether sunshine or rain would occur on the basis of one to three of the four visual cues. This task format is known as the *weather prediction task* (29) and has been used with various healthy and clinical populations for the assessment of PCL. On the emotional task variant, the *epidemic prediction task*, the cover story implied that the subject was situated in a public health department and was receiving information about the spreading of a new “deadly” virus from the health authorities, which had to be deciphered. Participants had to decide whether or not an epidemic virus infection was threatening on the basis of one to three of the four visual cues. The cover story and feedback stimuli used in the emotional prediction task were created to raise contamination- and responsibility-related OCD fears as these symptom subtypes/dimensions have been proven to be very common in OCD participants, appearing in about 75% of the OCD population (57).

To minimize exhaustion effects, the two PCL task variants were separately administered to each participant on two different test sessions approximately 1 week apart (7.6 ± 1.9 days). The order of task presentation was counterbalanced across participants. A response was scored as optimal (correct) response if the participant selected the outcome that was most strongly related to the cue pattern presented on that trial. The percentage of optimal responses scores reflected how well participants learned the cue–outcome associations. In accordance with the previous behavioral investigation (18), percentage of optimal responses for each task variant was analyzed in three blocks, each containing five units with 50 trials in total.

In addition to the active condition of PCL, a control condition called “hardware check” was administered. On each trial in this condition, a certain number of geometric shapes were presented and participants had to indicate whether the number of presented shapes was two or different from two. Feedback was given after each response. Each experimental run consisted of contiguous alternating units of the active condition (PCL) and the control condition (“hardware check”) with ten trials in each unit. The whole run contained 15 units of the PCL task and 15 units of the “hardware check” task, thereby including 30 units with 300 trials in total. Each unit was introduced by an instruction giving information that now the PCL task or the “hardware check” task had to be done. The order of the active and control condition was randomized across participants.

### Procedure

Before scanning, subjects briefly practiced five random PCL and five random control condition trials to familiarize them with the task requirements. Participants got introduced to the tasks by information presented on the screen prior to the practice trials. After the functional magnetic resonance imaging (fMRI) scanning, the neuropsychological and clinical data was assessed.

### Magnetic Resonance Imaging (MRI) Acquisition

Magnetic resonance imaging data were acquired using a 3-T MR scanner (Siemens TIM Trio, Erlangen, Germany) with a 12-channel head matrix receive coil at the Department of Psychiatry, University of Marburg. Functional images were obtained using a T2*-weighted gradient-echo echo-planar imaging sequence (EPI) sensitive to the Blood Oxygen Level Dependent (BOLD) contrast (38 slices, TR = 2,500 ms, TE = 30 ms, matrix size 64 × 64 voxels, field of view = 230 mm × 230 mm, in-plane resolution 3.59 mm × 3.59 mm, slice thickness 3.6 mm, gap size 0.36 mm, flip angle 90°, interleaved-ascending). Slices covered the whole brain and were positioned transaxially parallel to the intercommissural (AC-PC) plane. In each session, a total of 780 functional images were collected. For each subject, an additional high-resolution anatomical image was acquired using a T1-weighted magnetization-prepared rapid gradient-echo (3d MP-RAGE) sequence in sagittal plane (176 slices, TR = 1,900 ms, TE = 2.52 ms, matrix size 256 × 256 voxels, voxel size 1 mm × 1 mm × 1 mm, flip angle 9°).

### Data Analysis

#### Behavioral Data

Behavioral data (i.e., percentage of correct responses) were analyzed using a 2 × 2 × 3 ANOVA design with the between-subject factor Group (OCD participants vs. healthy controls) and the within-subjects factors Task (OCD-specific, neutral task) and Block (Blocks 1, 2, and 3) using the SPSS software package (IBM SPSS Statistics for Windows, 2012, Armonk, NY, USA). We expected a significant effect of Block, indicating successful learning across both experiments for both groups. We further hypothesized that participants with OCD as compared to healthy controls will show equal performance in the neutral task, but perform significantly worse in the OCD-specific task [Group × Task interaction; see Ref. (18)].

#### Imaging Data

Preprocessing and statistical analysis of the functional images were conducted using the SPM8 software package (Statistical Parametric Mapping, Welcome Trust Center for Neuroimaging, London, UK; http://www.fil.ion.ucl.ac.uk) and Matlab R2008b. For each subject, functional MRI data were analyzed separately for each session. The functional volumes were temporally and spatially realigned, normalized to Montreal Neurological Institute (MNI) space using the unified normalization-segmentation procedure of SPM8 (resulting voxel size 2 mm × 2 mm × 2 mm), and smoothed with an isotropic 8-mm full-width at half-maximum Gaussian kernel. Time series from each voxel were high-pass filtered (1/200-Hz cutoff) to remove low-frequency noise and signal drift.

Statistical analysis was performed in a two-level, mixed-effects procedure. At the first-level model, the preprocessed functional images of each subject and each session were submitted to a fixed-effect analysis, using the general linear model (GLM) at each voxel. The GLM model included three regressors. The first regressor modeled, as regressor of no interest, the instructions (combined for the PCL and the “hardware check” task). This regressor was explicitly modeled as an epoch regressor (i.e., with a duration > 0). The second and third regressors modeled the active condition (OCD-specific and neutral task, respectively) and the control condition (“hardware check” task). These regressors were modeled as event regressors (i.e., with a duration = 0) and were set at the presentation of the cue stimuli (see Figure 1). All regressors were convolved with the canonical hemodynamic response function employed by SPM8. The GLM model additionally included the time derivatives of the three epoch regressors as well as six regressors modeling head movement parameters. Parameter estimate (β−) images were calculated for each session and subject. As contrast of interest, we compared the parameter estimates of the PCL task (contrast weight 1) and the “hardware check” task (contrast weight −1). At the second level, the weighted parameter estimate images were combined using a flexible factorial design, with factors of subject, the between-subject factor *Group* (OCD participants vs. healthy controls), and the within-subject factor *Task* (OCD-specific vs. neutral task). In this random-effects model, we allowed for violations of sphericity by modeling non-independence across images from the same subject and unequal variances between conditions and subjects as implemented in SPM8. Anatomical localization of the activated brain regions was achieved using the Anatomy toolbox within SPM8 (58).

Our data analysis strategy comprised two steps. In a first step, we tested whether the paradigm elicited the same brain activation pattern as described in previous studies [e.g., Ref. (15)] by assessing brain activity for the neutral PCL task in healthy controls. We expected activations in the frontal and the occipital cortex, the cerebellum and the caudate nucleus, and deactivations in the medial temporal lobes (in particular the hippocampus) and the cingulate cortex. Accordingly, the contrast in the second-level model was set to [1 0 0 0 ones (1,10)/10] (and [−1 0 0 0 ones (1,10)/10], respectively) showing the activation (and deactivation, respectively).

In a second step, we tested hypotheses on neural differences between OCD participants and healthy controls (see Introduction). At first, we expected a significant effect of group. On the one hand, we assumed that the dysfunctions in the orbitofrontal–striatal system in OCD participants resulted in an aberrant (i.e., significantly lower or higher) recruitment of the striatum (caudate nucleus and putamen) in OCD participants during the PCL performance. On the other hand, we assumed that, based on the hypothesis that participants with OCD rather engage the explicit learning system, participants with OCD show a significantly higher activity than healthy controls in the hippocampus. Secondly, we expected a significant interaction of Group and Task. On the one hand, we assumed that participants with OCD show, as compared to healthy controls, specifically in the OCD-specific task altered (i.e., significantly lower or higher) activity in the striatum and higher activity in the hippocampus. In contrast, for healthy controls we did not expect differential activity between both tasks. On the other hand, we assumed, according to a previous study incorporating a fear-relevant PCL task (36), that only OCD participants show a reduced recruitment of the amygdala in the OCD-specific task. For the main effect of Group, we used the contrasts [1 1 −1 −1 ones (1,10)/10 −ones (1,10)/10] and [−1 −1 1 1 −ones (1,10)/10 ones (1,10)/10], for the interaction the contrasts [1 −1 −1 1] and [−1 1 1 −1].

Since we had specific anatomical hypotheses, data were analyzed using a region-of-interest (ROI) approach. As ROIs, we chose for the analysis of the main effect the bilateral striatum (caudate nucleus and putamen) and the bilateral hippocampus. For the analysis of the interaction effect, we additionally chose an ROI that incorporated both the hippocampus and the amygdala. All ROIs were created using the WFU pickatlas (dilation factor = 3) (59). Additionally, we also performed explorative whole-brain analyses.

With regard to statistical thresholds, we decided to present the statistical maps in all figures at *p* < 0.001 (uncorrected). This is, in our opinion, justified since the present study has, due to the small sample size, the character of a pilot study. For all resulting clusters however, we explicitly specified the voxel-level FWE-corrected threshold, as obtained by the Gaussian random field correction implemented in SPM8, in the corresponding tables. Using this procedure, we make explicitly clear which of the clusters are active at corrected thresholds. This procedure is based on recommendations outlined in Ref. (60), in which Poldrack and colleagues suggest that “if you have used an uncorrected threshold then state clearly that you have unquantified control of family-wise error. Corrected or both corrected and uncorrected inferences should be reported and clearly labeled according to the type of correction.” We want to make the reader also aware that the correctly corrected statistical threshold is not *p* < 0.05 (corrected), but has to be additionally corrected by a factor of 2 for analyses 1 and 2 (since we used a two-tailed statistical contrast, looking at the “activation” and “deactivation” pattern), and by a further factor of 2 for the ROI approach in analysis 2 (since we looked at both the contrast for the main effect and the interaction effect).

## Results

### Behavioral Results

Percentage of optimal responses in three blocks of 50 trials was analyzed for the two task variants and compared between groups. A 2 (Group) × 2 (Task) × 3 (Block) ANOVA comparing participants with OCD and healthy controls with repeated measures across Blocks 1 to 3 and across the two task variants yielded a significant effect of Block, *F*(2, 36) = 8.68, *p* = 0.001, $\eta_{p}^{2}$ = 0.325, indicating successful learning throughout the experiments. In contrast, we found no significant effect of Group, *F*(1, 18) = 0.036, *p* = 0.852, $\eta_{p}^{2}$ = 0.002, no significant effect of Task, *F*(1, 18) = 0.959, *p* = 0.340, $\eta_{p}^{2}$ = 0.051, no significant Task × Block × Group interaction, *F*(2, 36) = 0.186, *p* = 0.831, $\eta_{p}^{2}$ = 0.010, and no significant Group × Task interaction, *F*(1, 18) = 0.822, *p* = 0.377, $\eta_{p}^{2}$ = 0.044 (see Table S2 in Supplementary Material for means and SD per groups).

**Figure 2:**
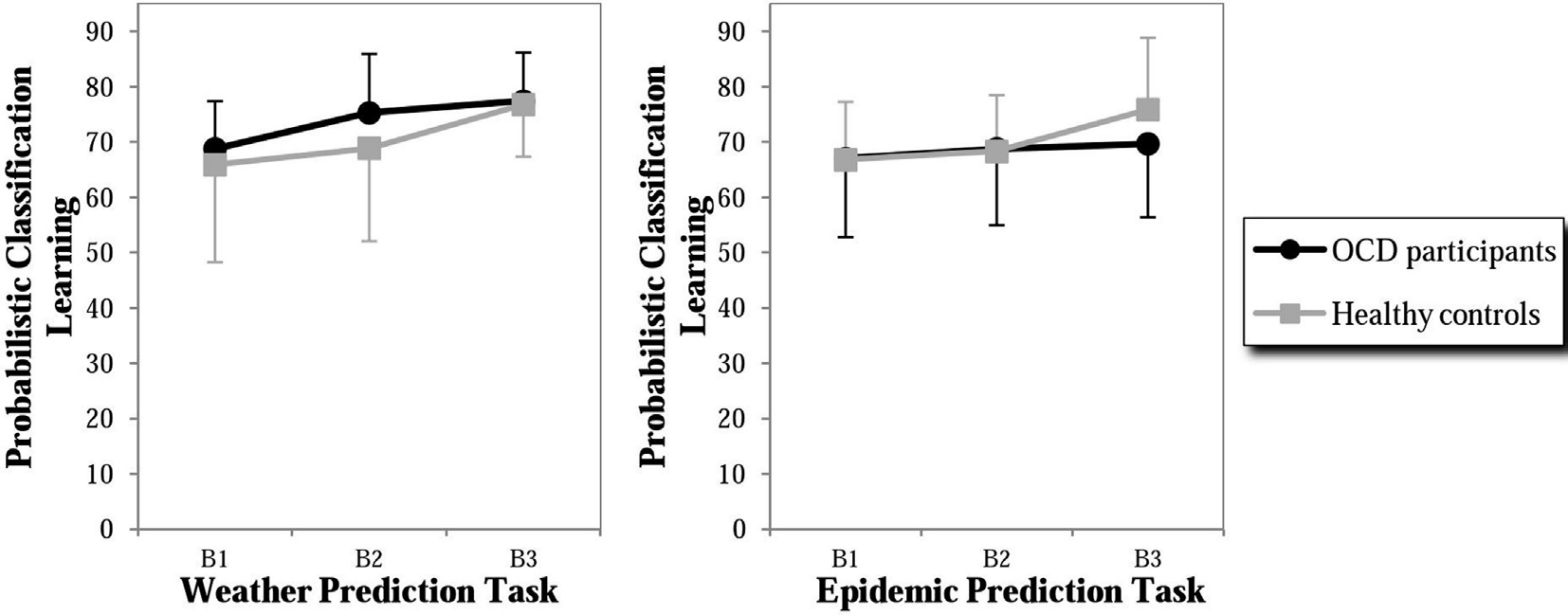
Percent of correct responses in probabilistic classification learning (PCL) of the weather prediction task (neutral content) and the epidemic prediction task (OCD-specific content) of participants with OCD and healthy controls. B1 = Block 1 of 50 trials, B2 = Block 2 of 50 trials, and B3 = Block 3 of 50 trials.

In order to analyze whether the findings of the previous study by Exner et al. (18) (using the same experimental design, assessing however only behavioral data) could be replicated on descriptive level in our study, additional analyses were performed. In the neutral task, both groups showed an increase of percentage of optimal responses across the three blocks with the OCD group performing slightly better than the control group (especially in Block 2, with effect size *d* = 0.46, Figure 2). The control group showed a similar performance in the OCD-specific task with an increasing percentage of optimal responses. In contrast, the OCD group showed only a minimal increase in the percentage of optimal response in the OCD-specific task in Block 3, leading to a medium-sized difference (effect size *d* = −0.47) between both groups. Hence, the control group showed a similar performance in both tasks, whereas participants with OCD failed to increase their classification accuracy during the last block in the OCD-specific task. In summary, the behavioral results of the present study were similar to those of our previous study, however without reaching significance, probably due to a smaller sample size in the present fMRI study.

### fMRI Results

In a first step, we assessed whether the same brain regions were activated in our study as in previous research using a PCL task with neutral material in healthy controls. In healthy controls, we found significant activation (contrast neutral PCL task > hardware check) bilaterally in the frontal, parietal, and the occipitotemporal cortex, the left cerebellum, the bilateral insula, the bilateral thalamus, and the striatum (right caudate nucleus and left putamen). For the opposite contrast (hardware check > neutral PCL task), we found significant “deactivation” in the anterior cingulate cortex, in the left frontal cortex, and the right occipital cortex (Figure S1 and Table S3 in Supplementary Material). These activation patterns were qualitatively similar to the activations reported in previous studies using PCL tasks [e.g., Ref. (15)], thus confirming the validity of our paradigm.

In a second step, we assessed functional differences between OCD participants and healthy controls.

#### Main Effect of Group

**Figure 3:**
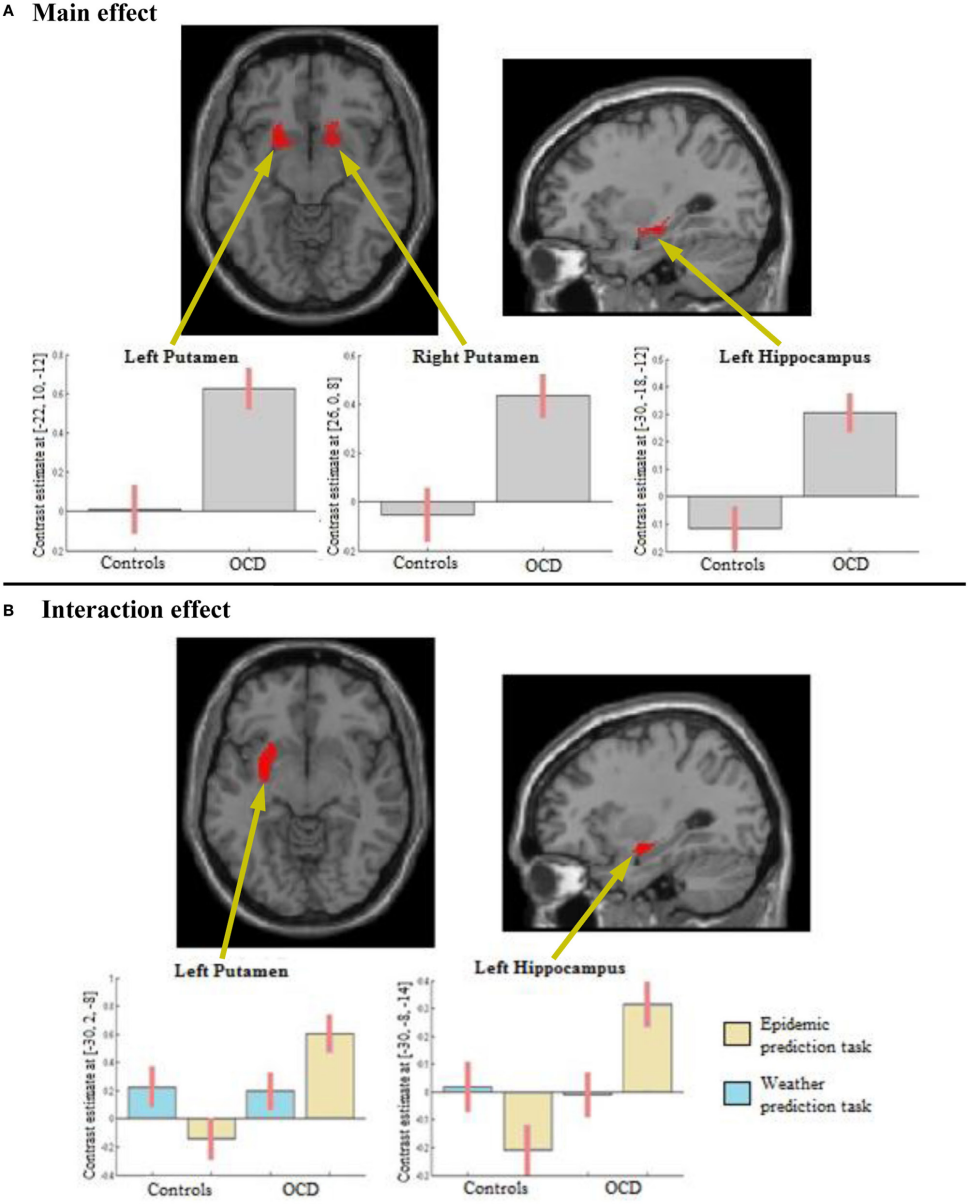
Results from the region of interest analyses. **(A)** Main effect of Group: obsessive-compulsive disorder (OCD) participants show higher activity than healthy controls in the left and right putamen and in the left hippocampus. **(B)** Interaction effect of Task (epidemic prediction task with OCD-specific content vs. weather prediction task with neutral content) and Group (participants with OCD vs. healthy controls): OCD participants show higher activity specifically in response to OCD-specific content in the left putamen and the left hippocampus.

In the ROI analyses, we found a main effect of group in the striatum, with participants with OCD showing a higher activity than healthy controls in the left and right putamen (Figure 3A; Table 1). We also found a main effect of group in the left hippocampus in the ROI analyses, again explained by higher activity of the OCD group compared to healthy controls (Figure 3A; Table 1). An explorative whole-brain analysis of the main effect of group showed that OCD participants had significantly more activation in the regions typically activated during PCL and less deactivation in the regions usually deactivated during PCL (Figure S2 and Table S4 in Supplementary Material). In contrast, no brain region was more activated in healthy controls than in OCD participants.

**Table 1:** Results from region of interest (ROI) analyses.

Location	*X*	*Y*	*Z*	*T*	*p*_FWE-corr_	Cluster size
**Activation of the main effect**						

**ROI striatum**						
L putamen	−22	12	−10	6.22	0.006	243
	−22	22	−6	6.17	0.006	
L putamen	−24	−12	−8	5.93	0.009	154
	−26	−22	2	5.87	0.011	
	−28	−20	−6	4.43	0.111	
R putamen	26	0	8	5.66	0.015	244
	28	8	8	5.06	0.039	
R putamen	16	6	−12	5.55	0.018	104
	20	26	−8	5.42	0.022	
	18	16	−8	4.12	0.178	
**ROI hippocampus**						
L hippocampus	−30	−18	−12	6.60	0.002	260
	−26	−10	−12	6.42	0.002	
	−10	−12	−22	5.12	0.021	

**Activation of the interaction effect**						

**ROI striatum**						
L putamen	−30	2	−8	5.33	0.026	182
	−26	14	−6	4.73	0.069	
	−28	−10	−8	4.23	0.150	
**ROI hippocampus and amygdala**						
L hippocampus	−30	−8	−14	6.20	0.004	103
	−30	−16	−12	4.48	0.071	

*Activation of the main (with obsessive-compulsive disorder participants showing higher activity than healthy controls) and the interaction effect with the ROIs striatum and hippocampus (and amygdala) (*p* < 0.001 on voxel level, cluster size > 100)*.

*L, left; R, right*.

#### Interaction Group × Task

In the ROI analyses, we found an interaction of Group × Task in the left putamen, explained by higher activation of the OCD group compared to healthy controls in the OCD-specific task but not the neutral task (Figure 3B; Table 1). We also found an interaction in the left hippocampus in the ROI analyses, again resulting from a higher activity especially in the OCD-specific task in OCD participants compared to healthy controls (Figure 3B; Table 1). An explorative whole-brain analysis of the interaction effect did mainly show activity differences in brain structures with significant differences found by the ROI analyses (Figure S3 and Table S5 in Supplementary Material).

## Discussion

The present study aimed to investigate neural correlates of implicit learning in OCD and extended prior findings by using an OCD-specific and a neutral task variant of the PCL paradigm. We found that (1) participants with OCD showed a significantly higher activity in the left and right putamen, but also in the left hippocampus during PCL compared to healthy controls, in the absence of significant differences in behavioral results of learning. In addition, (2) participants with OCD showed a significantly higher activity in the left putamen and in the left hippocampus especially in the OCD-specific task compared to healthy controls.

### Compensation of Striatal Deficits by Recruiting the Hippocampus

Considering that explicit memory depends on medial temporal lobe structures (with the hippocampus) and implicit memory on striatal circuitry (22), the present findings suggest that participants with OCD recruit both the striatum with the putamen and the hippocampus in the implicit learning task of PCL. An elevated activity of the striatum, also found during neutral states in OCD [e.g., Ref. (11)], might indicate a circuitry operating inefficiently and inflexibly. These findings are in good agreement with those obtained by structural and functional abnormalities in the orbitofrontal–striatal system frequently reported in OCD [e.g., Ref. (10)]. A deficient striatal activation in OCD was also found with regard to implicit learning in OCD (20).

Such a deficit in striatal circuits might be compensated by an enhanced activity in the medial temporal lobe to achieve adequate performance, as already indicated by previous studies (20, 21) showing an enhanced activity in medial temporal lobe structures in OCD. Consistent with this interpretation, participants with OCD were found to develop a greater explicit awareness of the embedded patterns in the implicit learning task SRT (5, 7). OCD participants exhibit a deficit in implicit learning if an explicit learning task has to be simultaneously performed (8). This might be due to the demands of the explicit learning tasks preventing them from using medial temporal lobe dependent strategies. The presumed compensation by explicit learning strategies in OCD participants might be similar to the shift in brain activation from the striatum to the medial temporal lobe found in patients with Parkinson’s disease (23), who have also shown to be impaired in implicit learning (61). The hypothesis of a recruitment of the medial temporal lobe in OCD participants might explain inconsistent findings regarding implicit learning performance in OCD participants, with some indicating a deficit [e.g., Ref. (6, 9)] and others not [e.g., Ref. (18, 19)]. Participants with OCD might sometimes be able to compensate for possible striatal deficits but might also fail in their attempt to recruit the medial temporal lobe for compensation.

Also our study supports the notion of a successful compensation by finding that participants with OCD, who show a higher activity in the striatum and the hippocampus than healthy controls, do not differ from them in behavioral results of PCL. However, with regard to the OCD-specific task, our study could show that, similar to the findings by Exner et al. (18), participants with OCD failed to improve their PCL performance contrary to healthy controls on trend level. Previous studies [e.g., Ref. (27, 28)] suggested an accentuated activity in cortico–thalamo–striatal pathways during symptom provocation in OCD participants, which might have also come into play during the OCD-specific task in OCD participants in our study. In consequence of the hyperactivation of the striatum, participants with OCD might have recruited the hippocampus extensively for compensation. Considering that the left hippocampus has been shown to be less active during PCL than during a control task in healthy controls in a previous study (15), the trial of compensation via the left hippocampus might have been detrimental, thereby leading to a content-related implicit learning deficit in OCD participants. In contrast, healthy controls show a deactivation of the left hippocampus in the OCD-specific task, which might have been related to an improvement in this PCL task variant. Interestingly, without showing a heightened activation of the striatum and the hippocampus, participants with OCD exhibit a better performance than healthy controls in the neutral task. It is important to note that the whole-brain analysis of the interaction effect did not show activity differences in further brain structures, thereby indicating that primarily the striatum and the hippocampus seem to be involved in differential performances of both groups in these task variants.

### Consequences of a Hippocampal Compensation

The effects of the overrecruitment of the hippocampus for the PCL performance might be mediated by its impact on learning strategies. Previous studies about strategies in PCL (62, 63) suggest that employing explicit learning strategies can affect the performance in some cases. Zetsche et al. (31) found that, using the same tasks as we did, OCD participants failed to adopt efficient learning strategies and showed fewer beneficial strategy switches than healthy controls only in the OCD-specific task. The number of beneficial strategy switches in the OCD-specific task mediated the difference in PCL performance between OCD participants and healthy controls. In healthy controls, arousal evoked by fearful outcomes in PCL was related to a suboptimal (34) or complex (36) strategy use. Hence, the possible overrecruitment of the hippocampus in response to a higher striatal activation might have led to an inflexible approach in learning during PCL and thereby to a deficit in implicit learning in OCD participants.

The consequences of the assumed processes during implicit learning may be detrimental for the maintenance of OC symptoms in different regards. At first, by employing hippocampus-based explicit learning in an implicit learning task, information usually processed implicitly (and thereby without awareness) probably gets consciously accessible. Our findings indicating that this particularly takes place in the OCD-specific task in OCD participants may be interpreted that OCD participants perceive threat-relevant information in the contexts of OCD-specific implicit learning situations with more awareness, which in turn may result in a constant feeling of being threatened by OCD-related risks, an evocation of intrusive and obsessional thoughts and thereby to an overestimation of threat. This interpretation would also be in line with the finding obtained by Zetsche, Rief, and Exner (30), showing that impairments of OCD participants in the OCD-specific PCL task were associated with their biases in the prediction of checking-related events. In addition, our findings, consistent with previous results (18), suggest a deficit of OCD participants in learning cue–outcome associations in OCD-specific contexts, which might result in misjudgments of the incidence of OCD-related events and thereby to a further exacerbation of biases of overestimation of threat. Hence, a vicious circle involving a conscious perception of threat-relevant information, deficits in learning cue–outcome associations in OCD-specific contexts, and an overestimation of threat may arise and contribute to the maintenance of OC symptoms. Further research is needed to determine whether these interpretations of our results are accurate.

### Involvement of the Amygdala

Contrary to our hypotheses, we did not find an aberrant activation of the amygdala in the OCD-specific task in OCD participants. Taking into account that the presentation of OCD-specific stimuli has been associated with exaggerated amygdala responses in OCD participants (12, 28, 64), an activation of the amygdala during the OCD-specific task in OCD participants would have been expected. However, with regard to PCL, the presence of fear-relevant outcomes was related to reduced recruitment of the amygdala in healthy controls in a previous study (36). The authors supposed that the presence of fear-relevant outcomes may have blunted the amygdala’s response to feedback incentives found in relation to neutral cues [e.g., Ref. (65)], thereby leading to the decreased activation of the amygdala. Accordingly, assuming that the prediction of a virus epidemic provoked OCD-specific fears in participants with OCD, the amygdala’s activity might have been decreased during the OCD-specific task in OCD participants in our study. This might have also tempered the possible exaggerated amygdala’s activity in response to the presentation of OCD-specific stimuli in OCD, resulting in a non-significant activation of the amygdala in our study. Hence, there might be an involvement of the amygdala in OCD-specific implicit learning in OCD participants, which might have not been found due to a mutual compensation of different effects in relation to the amygdala’s activation. Further research is needed to enlighten the role of this brain structure in implicit learning with disorder-relevant contents in OCD participants.

### Limitations

This study has several limitations. A modest number of subjects in both groups limit the informative value of the findings so that the conclusions remain somewhat speculative. The present study should be considered as a pilot experiment and future studies should replicate the current findings using a bigger sample size. In addition, the small sample size did not allow for differentiated analyses of learning performance across OCD subtypes. Different dimensions of OCD symptomatology were found to be associated with abnormalities in distinct components of the fronto-striatal circuitry (12) and, with regard to implicit learning, with differential activations of the striatum and the orbitofrontal cortex during the SRT in OCD participants (21). In addition, given that a previous study (9) suggests a subtle deficit in implicit learning in unmedicated patients with OCD, which may be mitigated by pharmacotherapy, the inclusion of participants with OCD being on psychotropic medication might have influenced our findings with respect to their performance and neural activity during the PCL task. Therefore, future studies should include unmedicated participants with OCD in a replication study of the present findings. In light of high rates of comorbidity in the OCD group, depression and anxiety symptoms might be a confounding factor. We therefore repeated the ANOVA analyses of behavioral results incorporating depression and anxiety symptoms as covariates, but did not find any significant effects. With regard to the cover story and stimuli of the epidemic prediction task, it remains unclear how much fear and arousal actually has been provoked in OCD participants. Although the cover story of this task was created to raise contamination- and responsibility-related OCD fears being highly frequent in OCD, these emotional contents did not encompass all individually relevant fears of participants with OCD in our sample. Previous findings (18, 30, 31), which show an impairment of participants with OCD in this task being related to an inflexible strategy use and biases in the prediction of checking-related events, support the claim of an emotional valence of this paradigm for OCD participants. However, including a measure of state arousal in future studies would be important to determine the extent of produced fear in OCD participants when working on this OCD-specific PCL task.

### Conclusion

In summary, the present study demonstrated an increased activation of both the putamen and the hippocampus in implicit learning in OCD participants especially in an OCD-specific task variant. This suggests that it is not a deficient basic cognitive process *per se* but rather an interplay of both an aberrant activation pattern and arousal being elicited by the OCD-specific task, which might account for a deviant behavioral performance in OCD participants. Taking into account that this may further nourish dysfunctional beliefs by a conscious perception of threat-relevant information and misjudgments in predicting OC-related events, a treatment addressing arousal in OCD-specific situations and beliefs of estimation of threat seems to be necessary to stop a further promotion of the development and maintenance of OCD symptoms. Cognitive behavioral therapy including a disputation of biased threat evaluations and exposure and response prevention fostering habituation to high arousal may be such a treatment approach. However, future research using OCD-specific material in learning and memory processes is required to elucidate the involved processes in information processes in OCD.

## Ethics Statement

This study was carried out in accordance with the recommendations of the last revision of the Declaration of Helsinki with written informed consent from all subjects. All subjects gave written informed consent in accordance with the Declaration of Helsinki. The protocol was approved by the Ethical Committee of the German Psychological Society (DGPs).

## Author Contributions

AJ, CE, and UZ conceived and designed the research experiment. UZ and JH performed the experiments. All authors interpreted the results of the experiments. AJ and JH drafted the manuscript and prepared figures. All authors edited and revised the manuscript and approved the final version of the manuscript.

## Conflict of Interest Statement

The authors declare that the research was conducted in the absence of any commercial or financial relationships that could be construed as a potential conflict of interest.
