# Supplementary figures and images for: The Neural Correlates of Probabilistic Classification Learning in Obsessive-Compulsive Disorder: A Pilot Study
Source: Front Psychiatry. 2018 Feb 28;9:58. doi: 10.3389/fpsyt.2018.00058 (PMC5863501; doi:10.3389/fpsyt.2018.00058)

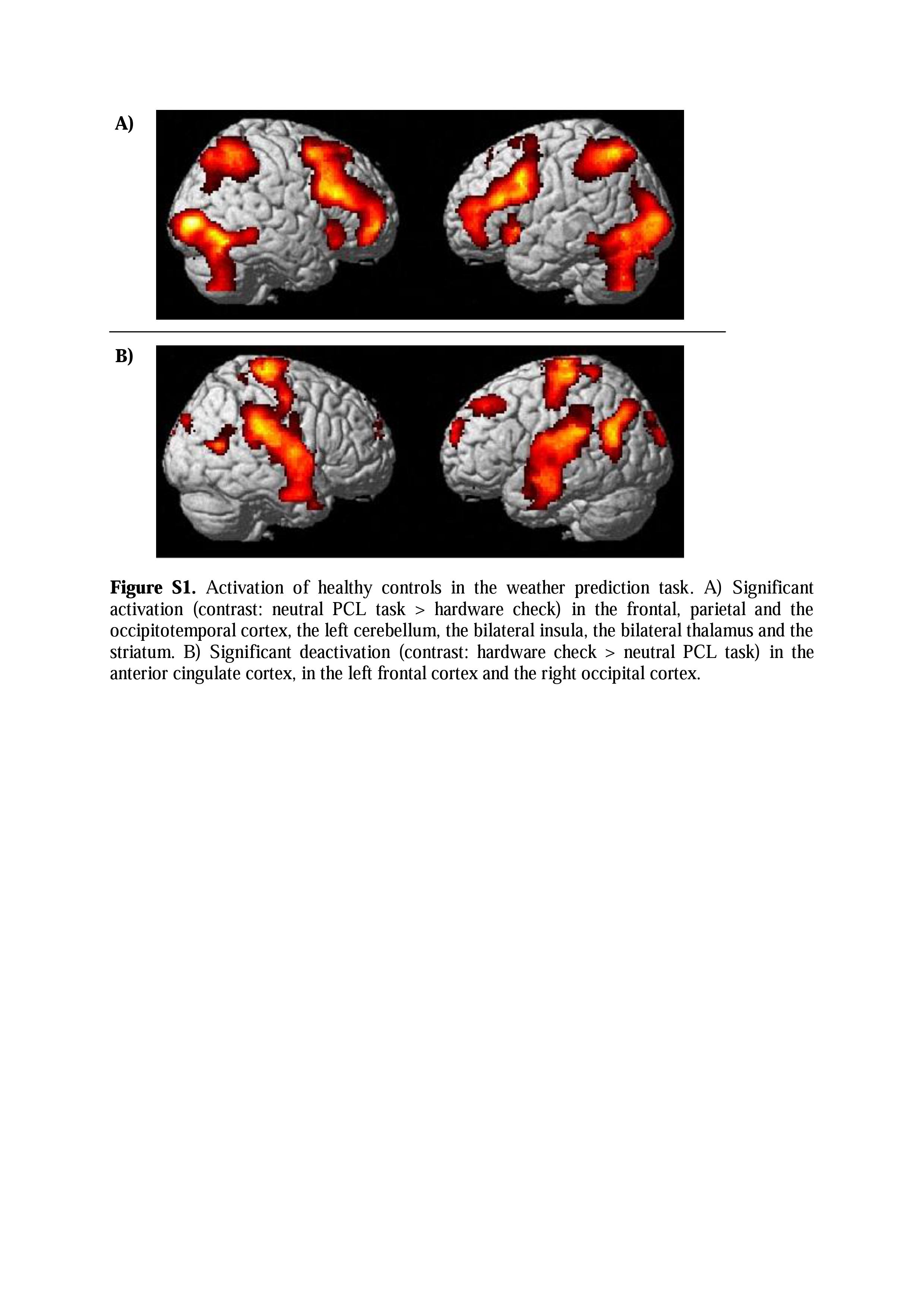

Supplement: Supplementary file 1 [file Image_1.jpeg]

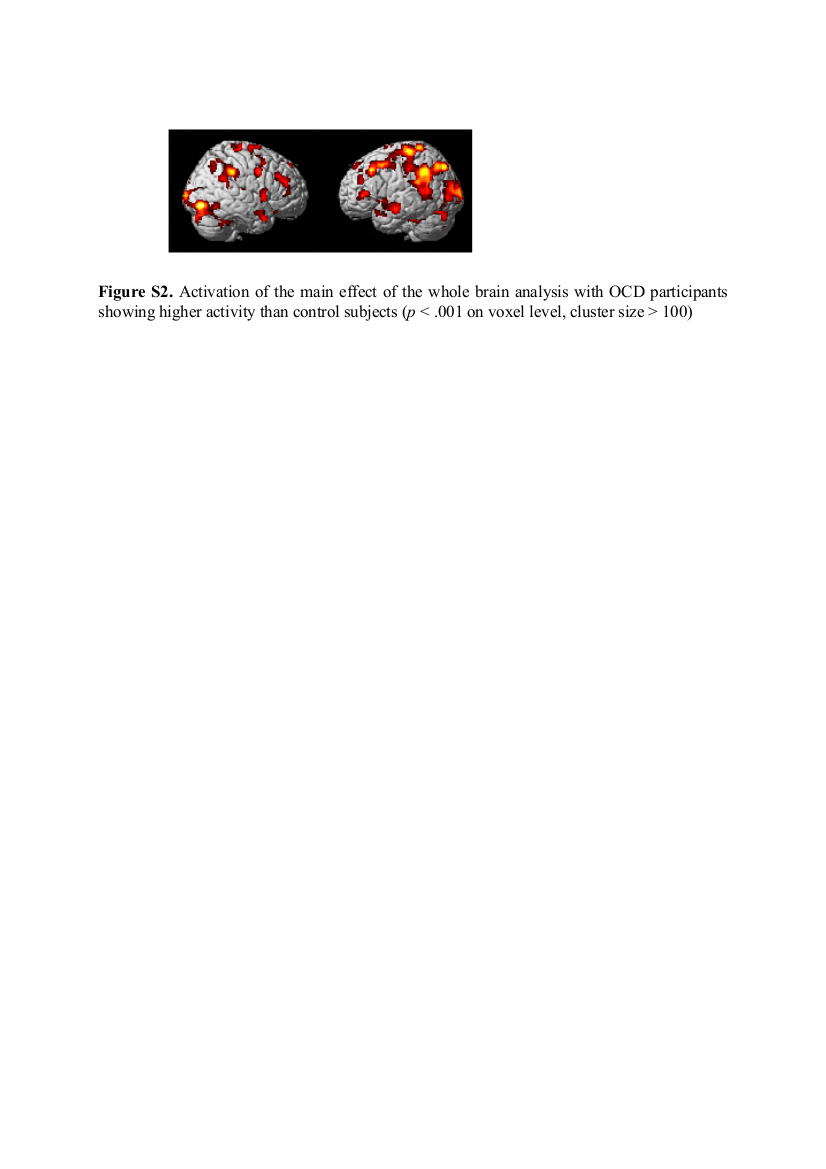

Supplement: Supplementary file 2 [file Image_2.jpg]

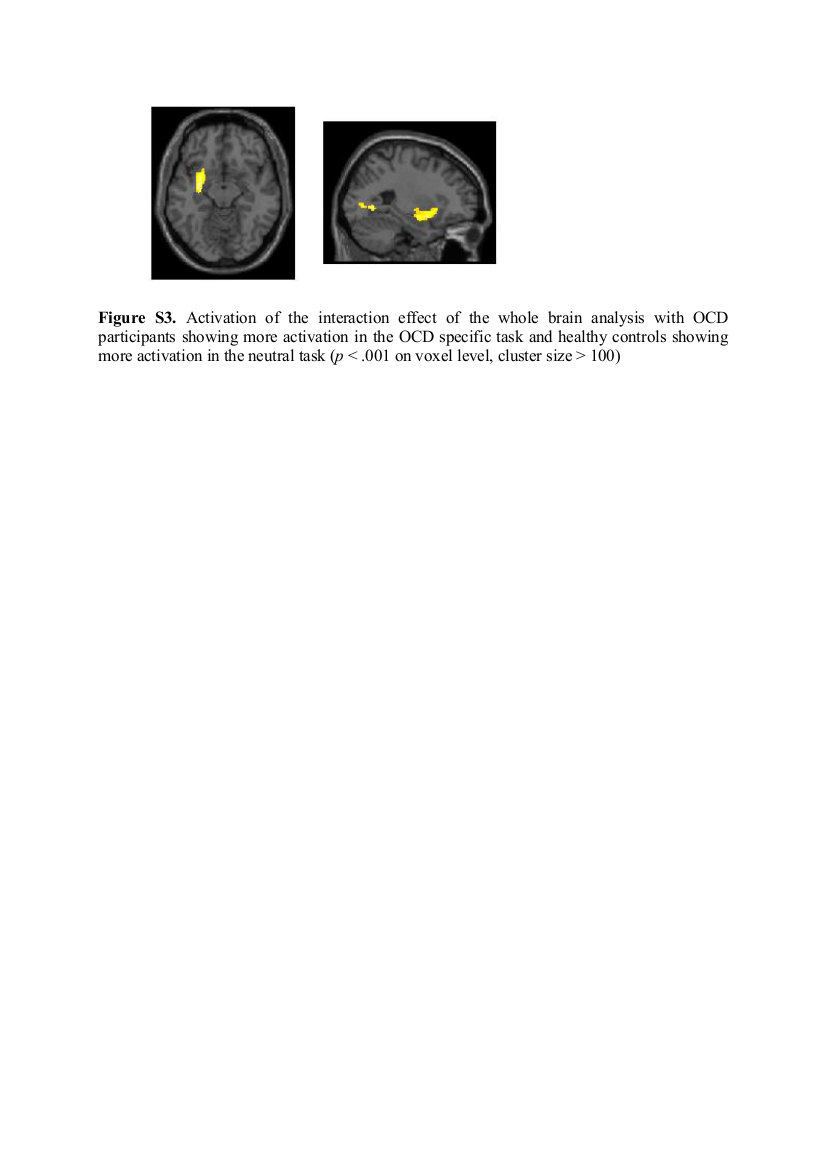

Supplement: Supplementary file 3 [file Image_3.jpg]
