# Supplementary material for: The Neural Correlates of Probabilistic Classification Learning in Obsessive-Compulsive Disorder: A Pilot Study
Source: Front Psychiatry. 2018 Feb 28;9:58. doi: 10.3389/fpsyt.2018.00058 (PMC5863501; doi:10.3389/fpsyt.2018.00058)
Supplement: Supplementary file 4 [file Table_1.docx]

**Table S1**

Demographic and clinical characteristics of participants

| Variable^a^ | | | Participants with OCD (*n* = 10) | Healthy Controls (*n* = 10) | Statistic | *p* |
| --- | --- | --- | --- | --- | --- | --- |
| **Age** | | | 29.7 ± 7.6 | 29.3 ± 8.6 | *t*(18) = -0.110 | 0.914 |
| **Education**^b^ | | | 15.3 ± 3.0 | 15.8 ± 2.2 | *t*(18) = -0.431 | 0.672 |
| **Gender**, no. (%) female | | | 6 (60.0) | 6 (60.0) | *χ*² = 0.000 | 1.000 |
|  | | |  |  |  |  |
| **WAIS-III**, scaled scores | | |  |  | *F*(4,15) = 0.706 | 0.600 |
|  | Information | | 11.5 ± 3.9 | 10.4 ± 2.6 | *F*(1,18) = 0.548 | 0.469 |
|  | Similarities | | 10.0 ± 2.0 | 8.7 ± 1.9 | *F*(1,18) = 2.233 | 0.152 |
|  | Picture Completion | | 8.8 ± 4.0 | 10.5 ± 4.0 | *F*(1,18) = 0.897 | 0.356 |
|  | Block Design | | 10.6 ± 3.5 | 9.7 ± 2.7 | *F*(1,18) = 0.423 | 0.524 |
|  |  | |  |  |  |  |
| **Duration of Disorder** | | | 10.0 ± 8.7 |  |  |  |
| **Y-BOCS**, Total Raw Score | | | 24.6 ± 5.1 |  |  |  |
|  | | Obsessions | 11.8 ± 2.3 |  |  |  |
|  | | Compulsions | 12.8 ± 3.0 |  |  |  |
|  | |  |  |  |  |  |
| **OBQ**, Total Raw Score | | | 151.3 ± 59.0 | 110.3 ± 32.6 | *t*(18) = -1.923 | 0.070 |
|  | | Importance of Thought | 22.9 ± 11.1 | 19.0 ± 6.1 | *t*(14.0) = -0.973^c^ | 0.347 |
|  | | Controls of Thoughts | 9.4 ± 4.6 | 7.7 ± 2.3 | *t*(13.1) = -1.044^c^ | 0.315 |
|  | | Perfectionism | 41.0 ± 18.0 | 28.7 ± 10.2 | *t*(14.2) = -1.883^c^ | 0.080 |
|  | | Intolerance of Uncertainty | 19.9 ± 8.2 | 14.3 ± 5.3 | *t*(18) = -1.817 | 0.086 |
|  | | Overestimation of Threat | 25.9 ± 13.5 | 15.1 ± 6.4 | *t*(12.8) = -2.289^c^ | **0.040** |
|  | | Inflated Responsibility | 32.2 ± 14.9 | 25.5 ± 9.4 | *t*(18) = -1.203 | 0.247 |
|  | |  |  |  |  |  |
| **BDI**, total raw score | | | 15.2 ± 14.1 | 5.3 ± 4.6 | *t*(10.9) = -2.103 ^c^ | 0.059 |
| **STAI**, total raw score | | | 2.3 ± 0.3 | 2.0 ± 0.2 | *t*(18) = -3.032 | **0.008** |

*WAIS-III*: Wechsler Adult Intelligence Scale, German Version; *BDI:* Beck Depression Inventory

^a^ Table values are given as mean ±S.D. unless indicated otherwise.

^b^ Number of years spent in full-time education.

^c^ Homogeneity correction in cases of heterogeneous variances.

Bold values refers to *p* < 0.05.
