# Supplementary material for: The Neural Correlates of Probabilistic Classification Learning in Obsessive-Compulsive Disorder: A Pilot Study
Source: Front Psychiatry. 2018 Feb 28;9:58. doi: 10.3389/fpsyt.2018.00058 (PMC5863501; doi:10.3389/fpsyt.2018.00058)
Supplement: Supplementary file 5 [file Table_2.docx]

**Table S2**

Behavioral data: Learning and memory performance

| Tests^a^ | Subscore | Participants with OCD | Healthy Controls | Statistic^b^ | *p* | Effect size d^c^ |
| --- | --- | --- | --- | --- | --- | --- |
| **Explicit Verbal Episodic Memory^d^** | |  |  | F(4,15) = 0.709 | 0.598 |  |
| **WMS-R** | **Logical Memory I** | 31.8 ± 7.0 | 30.6 ± 9.6 | F(1,18) = 0.102 | 0.753 | 0.14 |
|  | **Logical Memory II** | 26.7 ± 7.5 | 28.4 ± 7.8 | F(1,18) = 0.248 | 0.625 | -0.22 |
|  | **Verbal Paired Associates I** | 22.1 ± 1.6 | 22.2 ± 2.3 | F(1,18) = 0.012 | 0.913 | -0.05 |
|  | **Verbal Paired Associates II** | 7.9 ± 0.3 | 8.0 ± 0.0 | F(1,18) = 1.000 | 0.331 | -0.45 |
| **Explicit Visual Episodic Memory^d^** | |  |  | F(1,18) = 1.149 | 0.298 |  |
| **WMS-R** | **Visual Paired Associates I** | 16.7 ± 1.4 | 17.3 ± 1.1 | F(1,18) = 1.149 | 0.298 | -0.30 |
|  | **Visual Paired Associates II** | 6.0 ± 0.0 | 6.0 ± 0.0 | / | / | 0.00 |
|  |  |  |  |  |  |  |
| **Implicit Probabilistic Classification Learning** | | |  |  |  |  |
| **Weather Prediction Task** (neutral content) | |  |  | F(1, 18) = 0.59 | 0.451 |  |
| **% correct** | **Block 1 of 50 trials** | 68.8 ± 8.6 | 65.9 ± 17.6 |  |  | 0.21 |
|  | **Block 2 of 50 trials** | 75.3 ± 10.7 | 68.9 ± 16.8 |  |  | 0.46 |
|  | **Block 3 of 50 trials** | 77.4 ± 8.8 | 76.8 ± 9.4 |  |  | 0.07 |
|  |  |  |  |  |  |  |
| **Epidemic Prediction Task** (OCD-specific content) | |  |  | F(1, 18) = 0.13 | 0.725 |  |
| **% correct** | **Block 1 of 50 trials** | 67.0 ± 14.2 | 66.8 ± 10.5 |  |  | 0.02 |
|  | **Block 2 of 50 trials** | 68.8 ± 13.8 | 68.3 ± 10.2 |  |  | 0.05 |
|  | **Block 3 of 50 trials** | 69.7 ± 13.3 | 75.9 ± 13.0 |  |  | -0.47 |

*WMS-R:* Wechsler-Memory-Scale-Revised

^a^ Table values are given as mean ±S.D. unless indicated otherwise.

^b^ Between-subjects effects (see results section for complete statistics of multi-factor ANOVA models).

^c^ Effect sizes are group mean differences divided by pooled SD.

^d^ Multivariate ANOVA model across subtasks followed by univariate ANOVAS.
