# Supplementary material for: The Neural Correlates of Probabilistic Classification Learning in Obsessive-Compulsive Disorder: A Pilot Study
Source: Front Psychiatry. 2018 Feb 28;9:58. doi: 10.3389/fpsyt.2018.00058 (PMC5863501; doi:10.3389/fpsyt.2018.00058)
Supplement: Supplementary file 6 [file Table_3.docx]

**Table S3**

Activation (contrast: neutral PCL task > hardware check) and deactivation (contrast: hardware check > neutral PCL task) of the control group in the neutral PCL task (*p* < .001 on voxel level, cluster size > 250)

| Location | *X* | *Y* | *Z* | *T* | *p_FWE-corr_* | *Cluster Size* |
| --- | --- | --- | --- | --- | --- | --- |
| *Activation (contrast: neutral PCL task > hardware)* | | | | | | |
| R inferior occipital gyrus | 42 | -64 | -14 | 19.31 | 0.000 | 4701 |
| R middle occipital gyrus | 40 | -88 | 2 | 15.98 | 0.000 |  |
| R inferior occipital gyrus | 32 | -94 | -2 | 14.43 | 0.000 |  |
| L superior medial gyrus | -8 | 30 | 42 | 14.82 | 0.000 | 8067 |
| R middle frontal gyrus | 38 | 8 | 58 | 14.00 | 0.000 |  |
| R inferior frontal gyrus | 44 | 20 | 24 | 11.56 | 0.000 |  |
| L inferior frontal gyrus | -50 | 30 | 18 | 13.82 | 0.000 | 4930 |
| L inferior frontal gyrus | -46 | 10 | 28 | 13.07 | 0.000 |  |
| L middle frontal gyrus | -50 | 12 | 40 | 11.71 | 0.000 |  |
| L insula lobe | -32 | 18 | -6 | 13.31 | 0.000 | 839 |
| L cerebellum | -8 | -80 | -28 | 13.09 | 0.000 | 15849 |
| L middle occipital gyrus | -26 | -94 | 4 | 12.77 | 0.000 |  |
| L cerebellum | -32 | -70 | -50 | 12.72 | 0.000 |  |
| R insula lobe | 30 | 22 | -10 | 8.41 | 0.001 | 1011 |
| R insula lobe | 38 | 24 | 0 | 7.50 | 0.003 |  |
| L cingulate gyrus | -4 | -28 | 30 | 7.18 | 0.005 | 473 |
| R thalamus | 8 | -8 | 0 | 6.49 | 0.013 | 488 |
| R caudate nucleus | 12 | 12 | 2 | 4.71 | 0.195 |  |
| L thalamus | -12 | -8 | 0 | 6.04 | 0.026 | 439 |
| L caudate nucleus | -20 | -2 | 16 | 5.33 | 0.388 |  |
| L putamen | -14 | 10 | 0 | 4.51 | 0.836 |  |
|  |  |  |  |  |  |  |
| *Deactivation (contrast: hardware check > neutral PCL task)* | | | | | | |
| L mid orbital gyrus | -2 | 56 | -4 | 10.81 | 0.000 | 35571 |
| L midcingulate cortex | -10 | -28 | 46 | 9.98 | 0.001 |  |
| L anterior cingulate cortex | -10 | 34 | -8 | 9.92 | 0.001 |  |
| L superior frontal gyrus | -18 | 38 | 40 | 5.88 | 0.186 | 766 |
| L middle frontal gyrus | -28 | 30 | 40 | 5.63 | 0.264 |  |
| L superior medial gyrus | -8 | 52 | 44 | 4.72 | 0.725 |  |
| R superior occipital gyrus | 18 | -84 | 28 | 5.35 | 0.380 | 281 |
| R superior occipital gyrus | 22 | -80 | 22 | 4.99 | 0.567 |  |

*Abbreviations*: L – left, R – right.
