# Supplementary material for: The Neural Correlates of Probabilistic Classification Learning in Obsessive-Compulsive Disorder: A Pilot Study
Source: Front Psychiatry. 2018 Feb 28;9:58. doi: 10.3389/fpsyt.2018.00058 (PMC5863501; doi:10.3389/fpsyt.2018.00058)
Supplement: Supplementary file 7 [file Table_4.docx]

**Table S4**

Activation of the main effect of the whole brain analysis with OCD participants showing higher activity than control subjects (*p* < .001 on voxel level, cluster size > 100)

| Location | *X* | *Y* | *Z* | *T* | *p_FWEcorr_* | *Cluster Size* |
| --- | --- | --- | --- | --- | --- | --- |
| R inferior occipital gyrus | 40 | -80 | -12 | 13.47 | 0.000 | 2024 |
| L middle frontal gyrus | -36 | 22 | 36 | 11.83 | 0.000 |  |
| L middle frontal gyrus | -46 | 12 | 48 | 10.24 | 0.000 |  |
| L middle temporal gyrus | -58 | -4 | -14 | 7.48 | 0.018 | 318 |
| L middle temporal gyrus | -52 | -10 | -18 | 5.62 | 0.266 |  |
| Sub-Gyral/Parietal Lobe | 32 | -58 | 36 | 6.88 | 0.042 | 385 |
| R precentral gyrus | 46 | 2 | 38 | 6.43 | 0.083 | 438 |
| R precentral gyrus | 36 | 0 | 34 | 6.42 | 0.084 |  |
| R middle frontal gyrus | 44 | 8 | 54 | 4.99 | 0.571 |  |
| R supramarginal gyrus | 50 | -36 | 38 | 6.28 | 0.103 | 817 |
| R supramarginal gyrus | 62 | -40 | 42 | 6.21 | 0.116 |  |
| R supramarginal gyrus | 60 | -48 | 30 | 5.02 | 0.550 |  |
| L superior medial gyrus | -12 | 44 | 22 | 6.15 | 0.126 | 230 |
| R superior medial gyrus | 4 | 52 | 16 | 3.93 | 0.988 |  |
| R superior frontal gyrus | 24 | -10 | 74 | 5.92 | 0.175 | 108 |
| L middle frontal gyrus | -22 | 18 | 48 | 5.79 | 0.209 | 207 |
| Subcortical | -12 | 14 | 42 | 4.91 | 0.615 |  |
| RIFG (p. Orbitalis) | 26 | 28 | -14 | 5.70 | 0.237 | 305 |
| Sub-lobar/nucleus accumbens right | 16 | 6 | -12 | 5.55 | 0.290 |  |
| Sub-gyral/frontal lobe | 20 | 26 | -8 | 5.42 | 0.346 |  |
| R posterior-medial frontal | 6 | 4 | 74 | 5.70 | 0.237 | 109 |
| L posterior-medial frontal | -2 | -6 | 78 | 3.86 | 0.993 |  |
| R posterior-medial frontal | 6 | -6 | 66 | 3.74 | 0.997 |  |
| R putamen | 26 | 0 | 8 | 5.66 | 0.251 | 255 |
| R putamen | 28 | 8 | 8 | 5.06 | 0.527 |  |
| L middle frontal gyrus | -42 | 38 | 24 | 5.59 | 0.276 | 126 |
| L middle frontal gyrus | -36 | 40 | 32 | 4.78 | 0.691 |  |
| Precentral gyrus | 30 | -28 | 72 | 5.45 | 0.334 | 225 |
| R precentral gyrus | 20 | -24 | 78 | 3.94 | 0.987 |  |
| R middle frontal gyrus | 34 | 40 | 24 | 5.43 | 0.343 | 393 |
| R middle frontal gyrus | 44 | 30 | 34 | 5.07 | 0.523 |  |
| RIFG (p. Tirangularis) | 35 | 32 | 28 | 4.97 | 0.579 |  |
| R insula lobe | 48 | 8 | 6 | 5.32 | 0.393 | 260 |
| R middle temporal gyrus | 54 | 4 | -20 | 5.03 | 0.546 | 204 |
| R temporal pole | 40 | 12 | -30 | 4.78 | 0.691 |  |
| R medial temporal pole | 52 | 10 | -32 | 4.26 | 0.929 |  |
| L superior medial gyrus | -2 | 48 | 46 | 5.01 | 0.559 | 216 |
| L superior frontal gyrus | -16 | 38 | 36 | 4.45 | 0.862 |  |
| R posterior-medial frontal | 8 | 4 | 48 | 4.76 | 0.703 | 143 |
| R heschls gyrus | 42 | -20 | 12 | 4.65 | 0.767 | 192 |
| R insula lobe | 34 | -20 | 20 | 4.62 | 0.778 |  |

*Abbreviations*: L – left, R – right.
