# Supplementary material for: The Neural Correlates of Probabilistic Classification Learning in Obsessive-Compulsive Disorder: A Pilot Study
Source: Front Psychiatry. 2018 Feb 28;9:58. doi: 10.3389/fpsyt.2018.00058 (PMC5863501; doi:10.3389/fpsyt.2018.00058)
Supplement: Supplementary file 8 [file Table_5.docx]

**Table S5**

Activation of the interaction effect of the whole brain analysis with OCD participants showing more activation in the OCD specific task and healthy controls showing more activation in the neutral task (*p* < .001 on voxel level, cluster size > 100)

| Location | *X* | *Y* | *Z* | *T* | *p_FWEcorr_* | *Cluster Size* |
| --- | --- | --- | --- | --- | --- | --- |
| Sub-gyral/frontal lobe | 26 | -32 | 40 | 6.26 | 0.107 | 182 |
| Cingulate gyrus | 20 | -20 | 44 | 4.28 | 0.926 |  |
| Sub-gyral/frontal lobe | 26 | -36 | 28 | 4.26 | 0.932 |  |
| L hippocampus | -30 | -8 | -14 | 6.20 | 0.116 | 372 |
| Extra-nuclear/L putamen | -30 | 2 | -10 | 5.56 | 0.286 |  |
| L putamen | -26 | 14 | -6 | 4.73 | 0.723 |  |
| L lingual gyrus | -26 | -62 | -2 | 5.88 | 0.186 | 123 |
| Middle Occipital gyrus | -26 | -78 | 2 | 4.53 | 0.825 |  |

*Abbreviations*: L – left, R – right.
